# A systematic review of the clinical characteristics of influenza-COVID-19 co-infection

**DOI:** 10.1007/s10238-023-01116-y

**Published:** 2023-06-16

**Authors:** Karan Varshney, Preshon Pillay, Ashmit Daiyan Mustafa, Dennis Shen, Jenna Renee Adalbert, Malik Quasir Mahmood

**Affiliations:** 1 School of Medicine, Deakin University, 75 Pigdons Road, Waurn Ponds, VIC 3216 Australia; 2 School of Public Health and Preventive Medicine, Monash University, Melbourne, VIC Australia; 3 School of Medicine, University of New England, Armidale, NSW Australia; 4 University of Miami, Miami, FL USA

**Keywords:** COVID-19, Influenza, Co-infection, Pandemic, Vaccination, Pneumonia

## Abstract

COVID-19 has impacted populations across the globe and has been a major cause of morbidity and mortality. Influenza is another potentially deadly respiratory infection that affects people worldwide. While both of these infections pose major health threats, little is currently understood regarding the clinical aspects of influenza and COVID-19 co-infection. Our objective was to therefore provide a systematic review of the clinical characteristics, treatments, and outcomes for patients who are co-infected with influenza and COVID-19. Our review, which was conducted in accordance with the Preferred Reporting Items for Systematic Reviews and Meta-Analyses (PRISMA) guidelines, involved searching for literature in seven different databases. Studies were eligible for inclusion if they included at least one co-infected patient, were available in English, and described clinical characteristics for the patients. Data were pooled after extraction. Study quality was assessed using the Joanna Brigg’s Institute Checklists. Searches produced a total of 5096 studies, and of those, 64 were eligible for inclusion. A total of 6086 co-infected patients were included, 54.1% of whom were male; the mean age of patients was 55.9 years (SD = 12.3). 73.6% of cases were of influenza A and 25.1% were influenza B. 15.7% of co-infected patients had a poor outcome (death/deterioration). The most common symptoms were fever, cough, and dyspnea, with the most frequent complications being pneumonia, linear atelectasis, and acute respiratory distress syndrome. Oseltamivir, supplemental oxygen, arbidol, and vasopressors were the most common treatments provided to patients. Having comorbidities, and being unvaccinated for influenza, were shown to be important risk factors. Co-infected patients show symptoms that are similar to those who are infected with COVID-19 or influenza only. However, co-infected patients have been shown to be at an elevated risk for poor outcomes compared to mono-infected COVID-19 patients. Screening for influenza in high-risk COVID-19 patients is recommended. There is also a clear need to improve patient outcomes with more effective treatment regimens, better testing, and higher rates of vaccination.

**Supplementary Information:**

The online version contains supplementary material available at 10.1007/s10238-023-01116-y.

## Background

The severe acute respiratory syndrome coronavirus 2 (SARS-CoV-2) pandemic has swept the world since its discovery at the end of 2019, dominating health policy and economic activity to this day. Given the name coronavirus disease 19 (COVID-19) after its identification [1], there have been over 767 million confirmed COVID-19 cases globally over the course of the pandemic, exceeding 6.9 million deaths [2]. In contrast, annual seasonal influenza epidemics result in 290,000–650,000 deaths worldwide, despite affecting up to 20% of the population (depending on circulating viral strains) [3].

SARS-CoV-2 belongs to the coronavirus class of viruses [1], in contrast to the influenza class viruses of influenza, but both viruses share many structural similarities. Both are single-stranded enveloped RNA viruses, with influenza containing a negative-sense RNA strand [4] while coronaviruses are positive sense [1]. Both share similar infection sites, involving the upper respiratory tract (URT) and lower respiratory tracts (LRT). Influenza URT infections have high transmissibility but low virulence, while LRT infections can have more serious symptoms [4]. Additionally, both viruses share methods of transmission—namely droplet, aerosol, and self-inoculation via hand contamination—and both can be transmitted via human-to-human or indirect contact as a result of being in close contact [5]. Furthermore, COVID-19 presents with similar symptoms to influenza, where patients typically present with fevers, dyspnea, sore throat, rhinorrhea, myalgia, or nausea [3]. There are now vaccines for each of these viruses and are important in protection from infection.

Differences between COVID-19 and influenza arise from virulence characteristics and age groups affected. SARS-CoV-2 has a higher transmissibility (*R*_0_) rate and 2–10-day longer incubation period compared to influenza [6]. Moreover, COVID-19 mortality is skewed toward people > 70 years old, whereas the 1918 and 2009 influenza pandemics deaths were largely in people < 65 years old, and intensive care admission was 5–6 times fold higher compared to the influenza pandemic of 2009 [6]. Age of hospitalisation is similar across both viruses, with a study in France finding the median age of hospitalized COVID-19 patients being 68 years old, compared to 71 for influenza during the same period [7]. Importantly, while standardized antiviral treatment regimens do currently exist for influenza, this is not the case for COVID-19 despite the occurrence of numerous clinical trials. Hence, in order to prevent morbidity and mortality, it remains evident that preventative efforts against COVID-19 are integral, and new treatments are urgently needed. Considerably more research is also needed to better understand the molecular genetics and pathogenicity of COVID-19 as this will guide treatment development.

In regards to co-infections, the incidence rate of co-infection for COVID-19 with fungal and/or bacterial pathogens has been reported to be 8% and approximately 3% for other respiratory viruses in hospitalized patients [8, 9]. Overall, bacterial co-infections appear less prevalent in COVID-19 patients than those with influenza [9, 10], with the more common organisms being *Mycoplasma pneumoniae, Pseudomonas aeruginosa, Haemophilus influenzae* and* Klebisella pneumoniae* [9]. In contrast, bacteria associated with influenza co-infection are more likely to be those that colonize the nasopharynx, including *Streptococcus pneumoniae, Staphylococcus aureus,* and *Streptococcus pyogenes* [9]. In the 2009 influenza pandemic, a quarter of severe or fatal cases had a bacterial infection, affecting morbidity and mortality [9]. A recent study reported that while less than 1% of co-infected COVID-19 positive patients were found to have immunosuppression, they were nonetheless at a notably higher risk for additional infections and complications [11].

The clinical manifestations, treatment regimens, and outcomes for patients co-infected with COVID-19 and influenza are currently not well understood. While prior systematic reviews have been conducted on COVID-19 and influenza coinfection, they either had an exceedingly low number of included patients due to the review being conducted early in the pandemic [12], or they provided limited information regarding clinical presentations, complications, and treatment regimens [13]. As a result, there are still major gaps in the literature regarding the clinical aspects of COVID-19 and influenza co-infection. Thus, we have conducted a systematic review to identify the clinical characteristics of COVID-19 co-infection with influenza, and to explore its implications for clinical practice.

## Methods

This systematic review has been conducted in accordance with the Preferred Reporting Items for Systematic Review and Meta-Analyses (PRISMA) guidelines [14]. The following steps were followed in the process of conducting this review: (1) determination of the research topic and development of the research question, (2) development of search terms for respective databases, (3) conducting the searches in respective databases, (4) determining eligibility for inclusion of studies, (5) charting of data, (6) completion of quality assessments and determining relevant patterns in assessments, (7) pooling of data, and (8) analysing and reporting of findings.

On May 29, 2023, searches were conducted in OVID Medline, PubMed, Scopus, Cumulative Index of Nursing and Allied Health Literature (CINAHL), Global Health, Web of Science, and ScienceDirect. Searches were intended to encompass broad terms relating to COVID-19, influenza, and co-infection. There were no restrictions placed based on language, or date of publication. Reference lists of relevant articles were also screened to determine additional articles which may have eligible for inclusion. A full list of search terms, by database, is listed in Supplementary Table 1.

After searches were conducted, three researchers (KV, AM, and PP) each independently screened articles. First, duplicates were removed, and then articles were screened by title/abstract. Articles were then screened by full text to determine eligibility for our review. Discrepancies in articles selected after screening were resolved by consensus among reviewers.

In order to select as many relevant articles as possible, we aimed to keep our inclusion criteria as broad as possible. Studies were eligible for inclusion in this review if they:

- Involved original research (including preprints)
- Were available in English
- Included one or more patients co-infected with COVID-19 and influenza
- Described clinical characteristics of co-infected patients.

Characteristics of patient data were next charted from included articles. Aside from study design and country where the research was conducted, this review focused on patient demographics, health status, clinical manifestations, treatments, and outcomes. More precisely, data on number of patients, age, gender, influenza virus strain (A and/or B), comorbidities, symptoms, complications, vaccination status, treatments, and outcomes (recovered or death/deteriorated) were extracted. After charting of data was completed, data were pooled to show trends across the included studies.

Assessment of methodological quality for included studies was conducted using the Joanna Brigg Institute’s (JBI) tools for critical appraisal [15]. In a manner comparable to prior reviews [16, 17], the tools were modified to provide a numeric score based on the number of “yes” or “no” responses for each metric; as done previously by Adalbert et al. [18], scores were thereafter depicted visually to allow for comparisons. As per the JBI appraisal tools, cohort studies were on an eleven-item scale, both case reports and cross-sectional studies were on an eight-item scale, and both case series and case–control studies were on a ten-item scale.

## Results

**Fig. 1:**
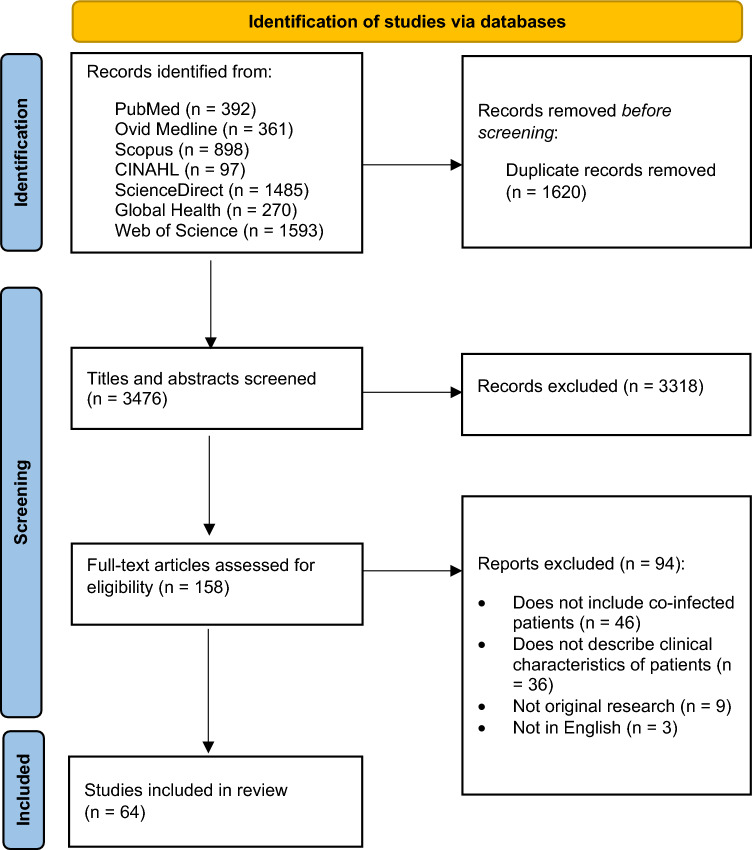
Process of searching and selecting articles included in the systematic review as per the PRISMA 2020 flow diagram (total results = 5096)

Initial searches across all databases produced a combined total of 5096 results. After removal of 1620 duplicates, 3476 articles were screened by title and abstract. A total of 158 articles were analysed by full text, 64 of which were deemed to be eligible for inclusion in this review [19–82]. The most common reasons for articles being excluded from this review were a lack of co-infected patients and a lack of a description of clinical characteristics. Figure 1 highlights the full screening process for this systematic review.

Studies in this review were conducted in 21 different countries. Studies were most frequently conducted in the USA (*n* = 18), China (*n* = 14), followed by Iran (*n* = 5) and India (*n* = 3). Two studies were conducted in each of the following countries: Saudi Arabia, Spain, UK, Turkey, Egypt, and Japan; a single study was conducted in each of the following countries: Italy, Peru, Germany, Bangladesh, Taiwan, Canada, South Korea, Poland, Greece, Brazil, and France. One study did not indicate the location [44]. The total number of co-infected patients across studies ranged from 1 to 4051. This review was composed of 29 case reports [19–47], 15 case series [48–62], 14 cohort studies [63–76], three cross-sectional studies [77–79], and three case–control studies [80–82]. Study characteristics are listed in Table 1.

**Table 1:** Characteristics of studies included in this systematic review

Study	Country	Study design	Total co-infection cases	Total cases with poor outcomes	Quality assessment score
Alhoufie [19]	Saudi Arabia	Case report	1	0	8/8
Azekawa [20]	USA	Case report	1	0	7/8
Baala [21]	France	Case report	1	1	6/8
Coutinho [22]	UK	Case report	1	0	8/8
D’Abramo [23]	Italy	Case report	1	0	7/8
Fahim [24]	Egypt	Case report	1	0	8/8
Farias [25]	Brazil	Case report	1	1	6/8
Hashemi [26]	Iran	Case report	2	2	5/8
Heshmat-Ghahdarijani [27]	Iran	Case report	2	0	6/8
Huang [28]	Taiwan	Case report	1	0	7/8
Hutto [29]	USA	Case report	1	0	8/8
Jing [30]	USA	Case report	1	0	8/8
Konala [31]	USA	Case report	1	Unspecified	7/8
Kondo [32]	Japan	Case report	1	0	8/8
Kwon [33]	USA	Case report	2	0	6/8
Lew [34]	USA	Case report	1	1	7/8
Lozano-Parras [35]	Spain	Case report	1	0	6/8
Maddali [36]	USA	Case report	1	0	6/8
Munivenkatappa [37]	India	Case report	1	1	8/8
Ning [38]	China	Case report	1	1	5/8
Ramalingam [39]	USA	Case report	1	0	5/8
Sang [40]	USA	Case report	1	1	5/8
Tomasik [41]	Poland	Case report	1	0	8/8
Tomos [42]	Greece	Case report	1	1	6/8
Valikhani [43]	Iran	Case report	1	0	8/8
Van Mecl [44]	Unspecified	Case report	1	0	6/8
Wehl [45]	Germany	Case report	1	0	6/8
Wu [46]	China	Case report	1	Unspecified	8/8
Xiang [47]	China	Case report	1	0	6/8
Agarwal [48]	India	Case series	9	3	7/10
Aggarwal [49]	India	Case series	5	0	9/10
Akhtar [50]	Bangladesh	Case series	5	1	8/10
Ali [51]	USA	Case series	5	1	6/10
Antony [52]	USA	Case series	3	0	10/10
Cuadrado-Payan [53]	Spain	Case series	4	0	7/10
Ding [54]	China	Case series	5	0	9/10
Kakuya [55]	Japan	Case series	1	0	9/10
Khodamoradi [56]	Iran	Case series	4	Unspecified	7/10
Konala [57]	USA	Case series	3	1	7/10
Miatech [58]	USA	Case series	4	0	8/10
Ozaras [59]	Turkey	Case series	6	0	10/10
Singh [60]	USA	Case series	3	0	3/10
Vargas-Ponce [61]	Peru	Case series	5	1	3/10
Zheng [62]	China	Case series	4	1	6/10
Adams [63]	USA	Cohort study	39	7	6/11
Alosaimi [64]	Saudi Arabia	Cohort study	17	5	7/11
Cheng [65]	China	Cohort study	97	9	9/11
Fahim [66]	Egypt	Cohort study	52	4	6/11
Islamoglu [67]	Turkey	Cohort study	87	Unspecified	8/11
Li [68]	China	Cohort study	3	Unspecified	7/11
Ma [69]	China	Cohort study	46	22	10/11
Roh [70]	South Korea	Cohort study	3	Unspecified	5/11
Schirmer [71]	USA	Cohort study	12	1	7/11
Stowe [72]	UK	Cohort study	56	25	8/11
Tong [73]	China	Cohort study	73	3	11/11
Wang [74]	China	Cohort study	151	15	7/11
Yue [75]	China	Cohort study	176	16	7/11
Zheng [76]	China	Cohort study	36	1	11/11
Hashemi [77]	Iran	Cross-sectional study	23	23	6/8
Peci [78]	Canada	Cross-sectional study	485	Unspecified	5/8
Tang [79]	China	Cross-sectional study	1	Unspecified	8/8
Garg [80]	USA	Case-control study	4501	706	9/10
Rizzo [81]	USA	Case-control study	58	5	7/10
Yu [82]	China	Case-control study	64	7	9/10

**Fig. 2:**
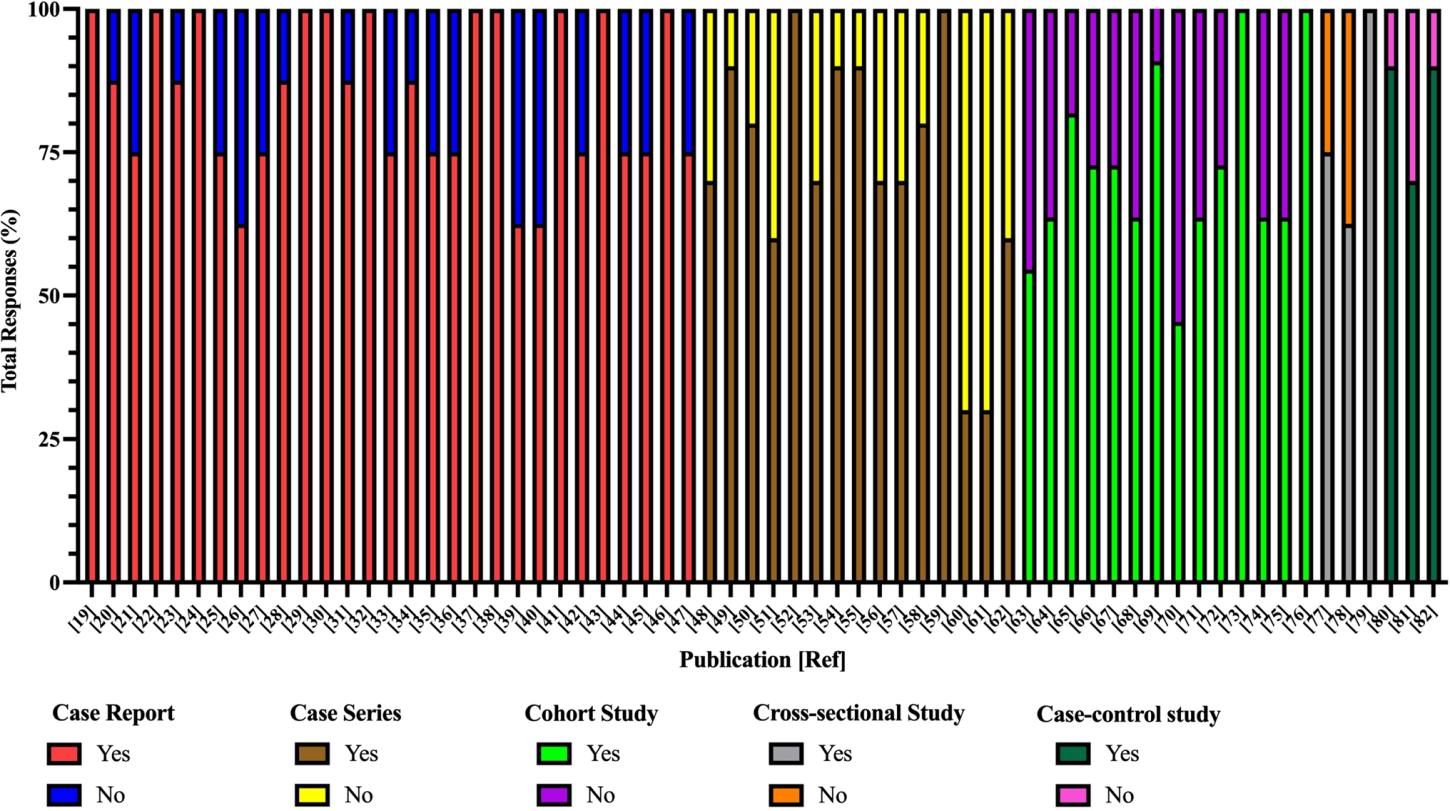
Quality assessment scores reported as “yes” or “no” for achieving quality metrics of included articles as per the Joanna Briggs Institute’s critical appraisal tools [15]

Mean quality assessment scores for the case reports were 6.79/8 (SD = 1.07), 7.27/10 for case series (SD = 2.15), 7.93/11 for cohort studies (SD = 1.75), 6.33/8 for cross-sectional studies (SD = 1.53), and 8.33/10 for case–control studies (SD = 1.15). Scores depictions (% yes/no) for quality assessments, by study, are listed in Fig. 2. The most common study design limitations, as per the quality assessments, were an inconsistency in reporting adverse events/complications, limited statistical analyses, a lack of consideration of confounding factors, and incomplete follow-up with limited strategies to address incomplete follow-up. Full quality assessment checklists are listed in Supplementary Tables 2–6.

Pooled characteristics of patients and their clinical manifestations are shown in Table 2. Across all studies, there was a combined total of 6086 co-infected patients included, with a mean age of 55.9 years (SD = 12.3). For studies in which gender was reported, it was shown that 45.9% of participants were female, and 54.1% were male. One study denoted that males had a 60% higher odds of becoming co-infected in comparison with females [78]. The majority of patients were co-infected with Influenza A (73.6%), whereas 25.1% were infected with influenza B. Strain of COVID-19 was not reported in any of the included studies. Comorbidities of patients were noted across studies, with the most common being hypertension (27.4%), diabetes (17.9%), obesity (11.4%) and chronic obstructive pulmonary disease (COPD) (10.1%). It was also relatively common for comorbidities to be unspecified (10.2%).

**Table 2:** Pooled characteristics of patients and clinical manifestations

Pooled factor	Count (%)
*Total patients (n* = *6086)*	
Gender (*n* = 5467)	
Male	2957 (54.1)
Female	2510 (45.9)
Mean age (SD)	55.9 years (= 12.3)
Strain of influenza (*n* = 1464)	
Influenza A	1077 (73.6)
Influenza B	367 (25.1)
Influenza A and B	20 (0.1)
Outcomes (*n* = 5491)	
Recovered	4628 (84.3)
Death/deterioration	863 (15.7)
Comorbidities (*n* = 10,391)	
Hypertension	2850 (27.4)
Diabetes	1862 (17.9)
Obese	1186 (11.4)
Unspecified comorbidity	1065 (10.2)
Chronic obstructive pulmonary disease	1046 (10.1)
Coronary artery disease/coronary heart disease	873 (8.4)
Heart failure	762 (7.3)
Past stroke	648 (6.2)
Overweight	18 (0.2)
Polyinfection	17 (0.2)
Chronic kidney disease	15 (0.1)
Malignancy	9 (0.09)
Liver disease	9 (0.09)
Asthma	9 (0.09)
Neurological disorder	6 (0.06)
Hyperlipidemia	5 (0.05)
Hypothyroidism	4 (0.04)
Underweight	3 (0.03)
Inflammatory condition	2 (0.02)
Past myocardial infarction	2 (0.02)
Symptoms (*n* = 1293)	
Fever	479 (37.0)
Cough	329 (25.4)
Dyspnea	175 (13.5)
Myalgia/fatigue	103 (8.0)
Diarrhea	50 (3.9)
Chest pain	43 (3.3)
Headache	30 (2.3)
Chills	28 (2.2)
Sore throat	10 (0.8)
Malaise	9 (0.7)
Nausea	7 (0.5)
Runny nose	6 (0.5)
Nasal congestion	6 (0.5)
Loss of appetite	4 (0.3)
Vomiting	4 (0.3)
Altered mental status	4 (0.3)
Orthopnea	3 (0.02)
Nocturnal sweats	1 (0.08)
Neck stiffness	1 (0.08)
Stridor	1 (0.08)
Complications (*n* = 226)	
Pneumonia	50 (22.1)
Linear atelectasis	46 (20.4)
Acute respiratory distress syndrome	40 (17.7)
Acute kidney injury	26 (11.5)
Cardiac arrest/acute cardiac injury	20 (8.8)
Fibrosis	16 (7.1)
Liver dysfunction	8 (3.5)
Lymphadenopathy	6 (2.7)
Hypoxemia	5 (2.2)
Bacteremia	3 (1.3)
Pleural effusion	3 (1.3)
Acute heart failure	3 (1.3)
Treatment (*n* = 1687)	
Oxygen/ventilation	1053 (62.4)
Arbidol	111 (6.6)
Oseltamivir	105 (6.2)
Vasopressor	103 (6.1)
Unspecified antibiotics	53 (3.1)
Unspecified antivirals	53 (3.1)
Hydroxychloroquine	32 (1.9)
Azithromycin	30 (1.8)
Unspecified glucocorticoids	27 (1.6)
Compound methoxamine capsule	17 (0.9)
Dexamethasone	15 (0.9)
Ceftriaxone	14 (0.8)
Unspecified vasoactive agents	13 (0.8)
Remdesivir	12 (0.7)
Anticoagulants	11 (0.7)
Methylprednisolone	9 (0.5)
Lopinavir	6 (0.4)
Enoxaparin	5 (0.3)
Ribavirin	5 (0.3)
Beta-1b 8MU	4 (0.2)
Vancomycin	3 (0.2)
Amoxicillin	2 (0.1)
Hydrocortisone	1 (0.06)
Doxycycline	1 (0.06)
Famotidine	1 (0.06)
Cefoperazaone	1 (0.06)
Vaccination history	
Most recent influenza vaccine	4/139 (2.9)
COVID-19 vaccine	1/3 (33.3)

Patients reported a wide range of symptoms on presentation. Fever (37.0%) and cough (25.4%) were among the most frequent, followed by dyspnea (13.5%), myalgia / fatigue (8.0%), and diarrhea (3.9%). Other reported symptoms were chest pain, headache, chills, malaise, sore throat, and nausea. Outcomes were commonly reported among hospitalized patients. Rates of poor outcomes (deterioration/death) were notably high, at 15.7%. In the study with the largest number of patients (4501), there was a total of 706 deaths reported (15.7%) [80]; the pooled death rate, when excluding the data from this study, was 15.9%. Stowe et al. [72] reported that co-infected patients were 2.27 times more likely to die in comparison with individuals infected with COVID-19 alone (95% CI, 1.23 to 4.19). Outcomes were reported far more consistently than complications, but complications also were reported in some studies; pneumonia (22.1%), linear atelectasis (20.4%), and acute respiratory distress syndrome (ARDS) (17.7%) were the most reported complications. Ma et al. reported stratified data among those who did not survive and demonstrated that, in this group, the most common complications which occurred prior to death were ARDS, acute kidney injury, acute cardiac injury, and liver dysfunction [69].

Treatment regimens frequently varied across the included studies. The most commonly reported form of support provided was oxygen/ventilation (62.4%). Aside from this, arbidol, oseltamivir, and vasopressors were the most frequently used forms of treatment. Antibiotics were also used in a notable number of cases; these antibiotics included azithromycin, ceftriaxone, vancomycin, amoxicillin, and doxycycline. Hydroxychloroquine was also utilized in some circumstances. Notably, vaccination status for COVID-19 was only described in three case report studies [25, 43, 49], and of the three cases, only one patient had received the COVID-19 vaccination. Influenza vaccination status in the most recent year was described across seven studies [22, 25, 31, 43, 49, 60] and a combined total of four out of 139 patients (2.9%) had received the vaccination.

## Discussion

In this systematic review, it was highlighted that hospitalized patients co-infected with influenza and COVID-19 are at a notably high risk of poor outcomes. Co-infected patients who exhibited major complications such as pneumonia, ARDS, and linear atelectasis are at a particularly high risk of mortality. Furthermore, individuals with comorbidities were shown to have high rates of co-infection; stratification for risk of mortality demonstrated that those with a wide array of differing comorbidities, such as hypertension, diabetes, obesity, COPD, coronary heart disease, heart failure, and past stroke are at an elevated risk of mortality. Therefore, this emphasizes a need to ensure protection of vulnerable individuals and that those who manifest clinical complications must be recognized as having a serious risk of further deterioration.

An important finding across this review was that the symptoms of COVID-19 and influenza co-infection are nearly identical to those of patients who are mono-infected with each one of these viruses. Considering the vast similarities of symptoms of co-infected and mono-infected patients, clinical suspicion alone is inadequate for identifying influenza in COVID-19-positive patients. To adequately identify the presence of influenza, screening high-risk COVID-19 patients in hospital settings will be integral. Furthermore, several patients received inadequate treatment regimens after being co-infected with these viruses, with some patients receiving antibiotics. Overall, this may have been a contributing factor for the high rates of poor outcomes among patients. This further emphasizes the need for screening of influenza in high-risk COVID-19 patients as it can lead to patients in poor conditions being managed with appropriate treatment regimens.

Critically, across the overwhelming majority of studies in this review, COVID-19 vaccination status was not considered for patients. As a result, there was no manner in which the influence of these vaccinations could be considered in regard to patient outcomes. However, influenza vaccination status was analysed across numerous studies. The findings of such studies showed that a very low number of those who had received the influenza vaccination (2.9%) had become co-infected. This finding has clear clinical implications and demonstrates that influenza vaccination is likely to provide protection against influenza-COVID-19 co-infection. Therefore, in consideration of the high rates of poor outcomes among co-infected patients, there is a clear need for interventions and programs to improve the uptake of influenza vaccinations. Kong et al. [83] highlight that the COVID-19 pandemic has helped to reduce influenza vaccine hesitancy, and hence serves as an important opportunity to further reduce hesitancy for this vaccine if appropriate efforts are made [83]. Therefore, public health programs and individual clinicians may be able to greatly reduce risk of both the incidence of co-infection, and the rates of poor outcomes, by promoting and recommending the influenza vaccination on an annual basis.

As well, there is also a clear and critical need for future research to further consider the potential impacts of both of these vaccinations on co-infected patient outcomes; large-scale case–control studies will be valuable to address this need, and it is recommended that healthcare providers screen co-infected patients for vaccination status in clinical settings to determine overall outcome risk. Alongside this research, there remains a need for future studies to better understand the genes and biological mechanisms of these viruses, and the manner with which they impact mortality risk when co-infection occurs. Such research can guide the development of treatments in the future [84], as well as the future development of combination vaccines for both influenza and COVID-19 [85]. For example, by determining networks of genes correlated with COVID-19 pathogenicity, Karami et al. [84] were able to identify 17 approved novel candidate drugs to be used to treat COVID-19 patients.

The findings of this review need to be considered alongside the limitations. While the studies included do provide significant insights on certain aspects of clinical manifestations, a notable number of these findings came from case series and case reports. There are hence limits to the generalizability of the findings and a need for large-scale cohort and case–control studies on co-infected patients. Furthermore, there were methodological limitations to this review. A limited number of databases were searched in this review, and eligible articles were restricted to those in English; therefore, it is possible that other relevant articles may have been missed during the screening process and that language bias may have had a role in this. As well, the broad inclusion criteria may have made the process of determining overall conclusions more difficult. As shown by the quality assessments, it is necessary for future studies to ensure long-term follow-up with patients and to aim to further understand the short- and long-term complications of co-infection. However, the limitations of the quality assessments themselves must be recognized, as the modified JBI checklists utilized across differing study methodologies may have not accounted for every aspect of overall study quality, such as bias and generalizability. It is also important to denote that there was limited stratification of factors relating to poor outcomes. As a result, there are limits to what can be stated regarding risk factors. Furthermore, the impacts of specific treatment regimens were not described in great detail across studies, and so specific recommendations regarding treatment cannot yet be made. More research is required regarding treatment regimens for co-infected patients.

Regardless of the limitations, our review also had notable strengths. In terms of methodology, studies were screened independently by three reviewers and included patients from a vast array of settings. Studies were conducted across 21 different countries and hence offer valuable insights regarding patients across diverse cultural and socioeconomic settings. Furthermore, with 64 studies included, the clinical manifestations have been described in detail across studies and can hence guide clinical practice. Our findings emphasize the importance of both screening for influenza in hospitalized COVID-19 patients and show the importance of promoting influenza vaccination uptake in the general population. Common symptoms, as well as important comorbidities and complications for co-infected patients, have been identified in this systematic review.

## Conclusions

Our review has shown that co-infected patients are at a notably high risk for poor outcomes, which may be higher than mono-infected patients. However, symptoms for co-infected patients are vastly similar to those of mono-infected patients, and this therefore emphasizes the importance of screening high-risk, COVID-19-positive patients for influenza in hospital settings. There is a clear need to improve patient outcomes with more effective treatment regimens, better testing, and higher rates of vaccination.

### Supplementary Information

Below is the link to the electronic supplementary material.

Supplementary file1 (DOCX 38 KB)
