# Supplementary material for: A systematic review of the clinical characteristics of influenza-COVID-19 co-infection
Source: Clin Exp Med. 2023 Jun 16;23(7):3265–75. doi: 10.1007/s10238-023-01116-y (PMC10618381; doi:10.1007/s10238-023-01116-y)
Supplement: Supplementary file 1 — Supplementary file1 (DOCX 38 KB) [file 10238_2023_1116_MOESM1_ESM.docx]

**Supplementary Table 1: Search terms used across different databases (searches conducted on May 29, 2023)**

| **Database (total results)** | **Search terms** |
| --- | --- |
| **PubMed (392)** | (severe acute respiratory syndrome coronavirus 2 [Title/Abstract] OR 2019 Novel coronavirus [Title/Abstract] OR 2019-nCoV infection [Title/Abstract] OR 2019 novel coronavirus infection [Title/Abstract] OR coronavirus disease 2019 virus [Title/Abstract] OR wuhan coronavirus [Title/Abstract] OR coronavirus [Title/Abstract] OR Novel coronavirus [Title/Abstract] OR coronavirus disease [Title/Abstract] OR covid 2019 [Title/Abstract] OR covid-19[Title/Abstract] OR sars-cov2 [Title/Abstract] OR 2019-ncov [Title/Abstract]) AND ("influenza, human"[MeSH] OR "Influenza B virus"[MeSH] OR "Influenza A virus"[MeSH] OR influenza[Title/Abstract] OR influenza A[Title/Abstract] OR influenza B[Title/Abstract] OR influenza C[Title/Abstract] OR influenza D[Title/Abstract]  OR flu[Title/Abstract]) AND ("Coinfection"[MeSH] OR co-infection[Title/Abstract] OR secondary infection[Title/Abstract] OR multiple infections[Title/Abstract]) |
| **Scopus (898)** | ( ( TITLE-ABS-KEY ( {severe acute respiratory syndrome coronavirus 2} ) OR TITLE-ABS-KEY ( {Novel coronavirus} ) OR TITLE-ABS-KEY ( {2019-nCoV infection} ) OR TITLE-ABS-KEY ( {2019 novel coronavirus infection} ) OR TITLE-ABS-KEY ( {coronavirus disease 2019 virus} ) OR TITLE-ABS-KEY ( {wuhan coronavirus} ) OR TITLE-ABS-KEY ( {coronavirus} ) OR TITLE-ABS-KEY ( {Novel coronavirus} ) OR TITLE-ABS-KEY ( {coronavirus disease} ) OR TITLE-ABS-KEY ( {covid 2019} ) OR TITLE-ABS-KEY ( {covid-19} ) OR TITLE-ABS-KEY ( {sars-cov2} ) OR TITLE-ABS-KEY ( {2019-ncov} ) ) ) AND ( ( TITLE-ABS-KEY ( {(influenza, human} ) OR TITLE-ABS-KEY ( {Influenza B virus} ) OR TITLE-ABS-KEY ( {Influenza A virus} ) OR TITLE-ABS-KEY ( {influenza} ) OR TITLE-ABS-KEY ( {influenza A} ) ) OR TITLE-ABS-KEY ( {influenza B} ) OR TITLE-ABS-KEY ( {influenza C} ) OR TITLE-ABS-KEY ( {influenza D} ) OR TITLE-ABS-KEY ( {flu} ) ) AND ( ( TITLE-ABS-KEY ( {(coinfection} ) OR TITLE-ABS-KEY ( {co-infection} ) OR TITLE-ABS-KEY ( {co-infection} ) OR TITLE-ABS-KEY ( {secondary infection} ) OR TITLE-ABS-KEY ( {multiple infections}) ) |
| **OVID Medline (361)** | (severe acute respiratory syndrome coronavirus 2 OR 2019 Novel coronavirus OR 2019-nCoV infection OR 2019 novel coronavirus infection OR coronavirus disease 2019 virus OR Novel coronavirus OR coronavirus disease OR covid-19 OR sars-cov2) AND (influenza, human OR Influenza B virus OR Influenza A virus OR influenza OR influenza A OR influenza B OR influenza C OR influenza D OR flu) AND (coinfection OR co-infection OR secondary infection OR multiple infections) |
| **CINAHL (97)** | (severe acute respiratory syndrome coronavirus 2 OR 2019 Novel coronavirus OR 2019-nCoV infection OR 2019 novel coronavirus infection OR coronavirus disease 2019 virus OR Novel coronavirus OR coronavirus disease OR covid-19 OR sars-cov2) AND (influenza, human OR Influenza B virus OR Influenza A virus OR influenza OR influenza A OR influenza B OR influenza C OR influenza D OR flu) AND (coinfection OR co-infection OR secondary infection OR multiple infections) |
| **ScienceDirect (1485)** | Articles with these terms: (influenza, human OR Influenza B virus OR Influenza A virus OR influenza OR influenza A OR influenza B OR influenza C OR influenza D OR flu)  Abs/Keywords: (coinfection OR co-infection OR secondary infection OR multiple infections) AND (severe acute respiratory syndrome coronavirus 2 OR 2019 Novel coronavirus OR coronavirus disease OR covid-19 OR sars-cov2) |
| **Global Health (270)** | (severe acute respiratory syndrome coronavirus 2 OR 2019 Novel coronavirus OR 2019-nCoV infection OR 2019 novel coronavirus infection OR coronavirus disease 2019 virus OR Novel coronavirus OR coronavirus disease OR covid-19 OR sars-cov2) AND (influenza, human OR Influenza B virus OR Influenza A virus OR influenza OR influenza A OR influenza B OR influenza C OR influenza D OR flu) AND (coinfection OR co-infection OR secondary infection OR multiple infections) |
| **Web of Science (1593)** | (severe acute respiratory syndrome coronavirus 2 OR 2019 Novel coronavirus OR 2019-nCoV infection OR 2019 novel coronavirus infection OR coronavirus disease 2019 virus OR Novel coronavirus OR coronavirus disease OR covid-19 OR sars-cov2) AND (influenza, human OR Influenza B virus OR Influenza A virus OR influenza OR influenza A OR influenza B OR influenza C OR influenza D OR flu) AND (coinfection OR co-infection OR secondary infection OR multiple infections) |

**Supplementary Table 2:** Joanna Briggs Quality Assessment for case reports included in the review

| First Author (Ref) | 1 | 2 | 3 | 4 | 5 | 6 | 7 | 8 | Total score |
| --- | --- | --- | --- | --- | --- | --- | --- | --- | --- |
| **Alhoufie [19]** | Yes | Yes | Yes | Yes | Yes | Yes | Yes | Yes | 8/8 |
| **Azekawa [20]** | Yes | Yes | Yes | Yes | Yes | Yes | No | Yes | 7/8 |
| **Baala [21]** | Yes | Yes | Yes | Yes | Yes | No | No | Yes | 6/8 |
| **Coutinho [22]** | Yes | Yes | Yes | Yes | Yes | Yes | Yes | Yes | 8/8 |
| **D’Abramo [23]** | Yes | Yes | Yes | Yes | Yes | No | Yes | Yes | 7/8 |
| **Fahim [24]** | Yes | Yes | Yes | Yes | Yes | Yes | Yes | Yes | 8/8 |
| **Farias [25]** | Yes | No | Yes | Yes | No | Yes | Yes | Yes | 6/8 |
| **Hashemi [26]** | No | Yes | Yes | Yes | Yes | No | No | Yes | 5/8 |
| **Heshmat-Ghahdarijani  [27]** | Yes | No | Yes | Yes | Yes | Yes | No | Yes | 6/8 |
| **Huang [28]** | Yes | Yes | Yes | Yes | Yes | Yes | No | Yes | 7/8 |
| **Hutto [29]** | Yes | Yes | Yes | Yes | Yes | Yes | Yes | Yes | 8/8 |
| **Jing [30]** | Yes | Yes | Yes | Yes | Yes | Yes | Yes | Yes | 8/8 |
| **Konala [31]** | Yes | Yes | Yes | Yes | Yes | Yes | Yes | No | 7/8 |
| **Kondo [32]** | Yes | Yes | Yes | Yes | Yes | Yes | Yes | Yes | 8/8 |
| **Kwon [33]** | Yes | Yes | Yes | Yes | Yes | No | Yes | No | 6/8 |
| **Lew [34]** | Yes | Yes | Yes | Yes | Yes | Yes | Yes | No | 7/8 |
| **Lozano-Parras [35]** | Yes | Yes | Yes | Yes | No | Yes | Yes | Yes | 6/8 |
| **Maddali [36]** | Yes | Yes | Yes | Yes | No | No | Yes | Yes | 6/8 |
| **Munivenkatappa [37]** | Yes | Yes | Yes | Yes | Yes | Yes | Yes | Yes | 8/8 |
| **Ning [38]** | Yes | Yes | Yes | Yes | Yes | Yes | Yes | Yes | 8/8 |
| **Ramalingam [39]** | Yes | No | Yes | Yes | No | Yes | No | Yes | 5/8 |
| **Sang [40]** | Yes | No | No | Yes | No | Yes | Yes | Yes | 5/8 |
| **Tomasik [41]** | Yes | Yes | Yes | Yes | Yes | Yes | Yes | Yes | 8/8 |
| **Tomos [42]** | Yes | No | Yes | Yes | Yes | Yes | No | Yes | 6/8 |
| **Valikhani [43]** | Yes | Yes | Yes | Yes | Yes | Yes | Yes | Yes | 8/8 |
| **Van Mecl [44]** | Yes | Yes | No | No | Yes | Yes | Yes | Yes | 6/8 |
| **Wehl [45]** | No | Yes | Yes | Yes | Yes | No | Yes | Yes | 6/8 |
| **Wu [46]** | Yes | Yes | Yes | Yes | Yes | Yes | Yes | Yes | 8/8 |
| **Xiang [47]** | Yes | No | Yes | Yes | Yes | Yes | No | Yes | 6/8 |

1. Were the patient’s demographic characteristics clearly described?
2. Was the patient’s history clearly described and presented as a timeline?
3. Was the current clinical condition of the patient on presentation clearly described?
4. Were diagnostic tests or assessment methods and the results clearly described?
5. Was the intervention(s) or treatment procedure(s) clearly described?
6. Was the post-intervention clinical condition clearly described?
7. Were adverse events (harms) or unanticipated events identified and described?
8. Does the case report provide takeaway lessons?

**Supplementary Table 3:** Joanna Briggs Quality Assessment for case-series included in the review

| First Author (Ref) | 1 | 2 | 3 | 4 | 5 | 6 | 7 | 8 | 9 | 10 | Total Score |
| --- | --- | --- | --- | --- | --- | --- | --- | --- | --- | --- | --- |
| **Agarwal [48]** | Yes | Yes | Yes | Yes | No | Yes | Yes | Yes | No | No | 7/10 |
| **Aggarwal [49]** | Yes | Yes | Yes | Yes | Yes | Yes | Yes | Yes | Yes | No | 9/10 |
| **Akhtar [50]** | Yes | No | Yes | Yes | Yes | Yes | Yes | Yes | No | Yes | 8/10 |
| **Ali [51]** | No | Yes | Yes | No | No | Yes | Yes | Yes | Yes | No | 6/10 |
| **Antony [52]** | Yes | Yes | Yes | Yes | Yes | Yes | Yes | Yes | Yes | Yes | 10/10 |
| **Cuadrado-Payan [53]** | Yes | Yes | Yes | No | No | Yes | Yes | Yes | Yes | No | 7/10 |
| **Ding [54]** | Yes | Yes | Yes | Yes | Yes | Yes | Yes | Yes | Yes | No | 9/10 |
| **Kakuya [55]** | Yes | Yes | Yes | Yes | Yes | Yes | Yes | Yes | Yes | No | 9/10 |
| **Khodamoradi [56]** | Yes | Yes | Yes | Yes | Yes | Yes | No | No | Yes | No | 7/10 |
| **Konala [57]** | Yes | Yes | Yes | No | No | Yes | Yes | Yes | Yes | No | 7/10 |
| **Miatech [58]** | No | No | Yes | Yes | Yes | Yes | Yes | Yes | Yes | Yes | 8/10 |
| **Ozaras [59]** | Yes | Yes | Yes | Yes | Yes | Yes | Yes | Yes | Yes | Yes | 10/10 |
| **Singh [60]** | No | No | Yes | No | No | Yes | Yes | No | No | No | 3/10 |
| **Vargas-Ponce [61]** | No | No | Yes | No | No | Yes | Yes | No | No | No | 3/10 |
| **Zheng [62]** | Yes | Yes | Yes | Yes | Yes | Yes | No | No | No | No | 6/10 |

1. Were there clear criteria for inclusion in the case series?
2. Was the condition measured in a standard, reliable way for all participants included in the case series?
3. Were valid methods used for identification of the condition for all participants included in the case series?
4. Did the case series have consecutive inclusion of participants?
5. Did the case series have complete inclusion of participants?
6. Was there clear reporting of the demographics of the participants in the study?
7. Was there clear reporting of clinical information of the participants?
8. Were the outcomes or follow up results of cases clearly reported?
9. Was there clear reporting of the presenting site(s)/clinic(s) demographic information?
10. Was statistical analysis appropriate?

**Supplementary Table 4:** Joanna Briggs Quality Assessment for cohort studies included in the review

| First Author (Ref) | 1 | 2 | 3 | 4 | 5 | 6 | 7 | 8 | 9 | 10 | 11 | Total score |  |
| --- | --- | --- | --- | --- | --- | --- | --- | --- | --- | --- | --- | --- | --- |
| **Adams [63]** | Yes | Yes | Yes | No | No | Yes | Yes | No | No | No | Yes | 6/11 |  |
| **Alosaimi [64]** | Yes | No | Yes | Yes | Yes | Yes | Yes | No | No | No | Yes | 7/11 |  |
| **Cheng [65]** | Yes | Yes | Yes | Yes | No | Yes | Yes | Yes | Yes | No | Yes | 9/11 |  |
| **Fahim [66]** | Yes | Yes | Yes | No | No | Yes | Yes | No | Yes | No | No | 6/11 |  |
| **Islamoglu [67]** | Yes | Yes | Yes | Yes | Yes | Yes | Yes | No | No | No | Yes | 8/11 |  |
| **Li [68]** | Yes | Yes | Yes | Yes | No | Yes | Yes | No | No | No | Yes | 7/11 |  |
| **Ma [69]** | Yes | Yes | Yes | Yes | No | Yes | Yes | Yes | Yes | Yes | Yes | 10/11 |  |
| **Roh [70]** | Yes | Yes | Yes | No | No | Yes | No | No | No | No | Yes | 5/11 |  |
| **Schirmer [71]** | Yes | Yes | Yes | No | No | Yes | Yes | Yes | No | No | Yes | 7/11 |  |
| **Stowe [72]** | Yes | Yes | Yes | Yes | Yes | Yes | Yes | No | No | No | Yes | 8/11 |  |
| **Tong [73]** | Yes | Yes | Yes | Yes | Yes | Yes | Yes | Yes | Yes | Yes | Yes | 11/11 |  |
| **Wang [74]** | Yes | Yes | Yes | No | No | Yes | Yes | No | No | No | Yes | 7/11 |  |
| **Yue [75]** | Yes | Yes | Yes | Yes | No | Yes | Yes | No | No | No | Yes | 7/11 |  |
| **Zheng [76]** | Yes | Yes | Yes | Yes | Yes | Yes | Yes | Yes | Yes | Yes | Yes | 11/11 |  |

1. Were the two groups similar and recruited from the same population?
2. Were the exposures measured similarly to assign people to both exposed and unexposed groups?
3. Was the exposure measured in a valid and reliable way?
4. Were confounding factors identified?
5. Were strategies to deal with confounding factors stated?
6. Were the groups/participants free of the outcome at the start of the study (or at the moment of exposure)?
7. Were the outcomes measured in a valid and reliable way?
8. Was the follow up time reported and sufficient to be long enough for outcomes to occur?
9. Was follow up complete, and if not, were the reasons to loss to follow up described and explored?
10. Were strategies to address incomplete follow up utilized?
11. Was appropriate statistical analysis used?

**Supplementary Table 5:** Joanna Briggs Quality Assessment for analytical cross-sectional studies included in the review

| First Author (Ref) | 1 | 2 | 3 | 4 | 5 | 6 | 7 | 8 | Total score |
| --- | --- | --- | --- | --- | --- | --- | --- | --- | --- |
| **Hashemi [77]** | Yes | Yes | Yes | Yes | Yes | No | Yes | No | 6/8 |
| **Peci [78]** | Yes | Yes | Yes | Yes | No | No | No | Yes | 5/8 |
| **Tang [79]** | Yes | Yes | Yes | Yes | Yes | Yes | No | Yes | 8/8 |

1. Were the criteria for inclusion in the sample clearly defined?
2. Were the study subjects and the setting described in detail?
3. Was the exposure measured in a valid and reliable way?
4. Were objective, standard criteria used for measurement of the condition?
5. Were confounding factors identified?
6. Were strategies to deal with confounding factors stated?
7. Were the outcomes measured in a valid and reliable way?
8. Was appropriate statistical analysis used?

**Supplementary Table** **6:** Joanna Briggs Quality Assessment for case control studies included in the review

| First Author (Ref) | 1 | 2 | 3 | 4 | 5 | 6 | 7 | 8 | 9 | 10 | Total Score |
| --- | --- | --- | --- | --- | --- | --- | --- | --- | --- | --- | --- |
| **Garg [80]** | Yes | Yes | Yes | Yes | Yes | Yes | No | Yes | Yes | Yes | 9/10 |
| **Rizzo [81]** | Yes | Yes | Yes | Yes | Yes | Yes | No | Yes | No | No | 7/10 |
| **Yu [82]** | Yes | Yes | Yes | Yes | Yes | Yes | No | Yes | ­­Yes | Yes | 9/10 |

1. Were the groups comparable other than the presence of disease in cases or the absence of disease in controls?
2. Were cases and controls matched appropriately?
3. Were the same criteria used for identification of cases and controls?
4. Was exposure measured in a standard, valid and reliable way?
5. Was exposure measured in the same way for cases and controls?
6. Were confounding factors identified?
7. Were strategies to deal with confounding factors stated?
8. Were outcomes assessed in a standard, valid and reliable way for cases and controls?
9. Was the exposure period of interest long enough to be meaningful?
10. Was appropriate statistical analysis used?
